# Lateralized brain connectivity in auditory verbal hallucinations: fMRI insights into the superior and middle temporal gyri

**DOI:** 10.3389/fnhum.2025.1650178

**Published:** 2025-08-20

**Authors:** Vyara Zaykova, Sevdalina Kandilarova, Rositsa Paunova, Ferihan Popova, Drozdstoy Stoyanov

**Affiliations:** ^1^Department of Anatomy, Histology and Embryology, Faculty of Medicine, Medical University of Plovdiv, Plovdiv, Bulgaria; ^2^Department of Psychiatry and Medical Psychology, Research Institute and SRIPD-MUP, Translational and Computation Neuroscience Group, Faculty of Medicine, Medical University of Plovdiv, Plovdiv, Bulgaria

**Keywords:** functional connectivity, schizophrenia, auditory verbal hallucinations, resting-state, functional magnetic resonance imaging, superior temporal gyrus, middle temporal gyrus

## Abstract

**Introduction:**

Auditory verbal hallucinations are one of the most prevalent positive symptoms associated with schizophrenia. The superior and middle temporal gyri have been demonstrated to play a role in auditory and language perception. Dysfunction in the temporal cortex has been associated with the development of psychosis. The aim of the present study was to explore the functional connectivity and laterality of superior and middle temporal gyri in patients with auditory verbal hallucinations.

**Methods:**

Resting-state functional magnetic resonance imaging data was obtained from a total of 105 subjects including 63 healthy controls and 42 patients diagnosed with schizophrenia experiencing auditory verbal hallucinations. A comparative analysis was conducted to assess the functional connectivity of the superior and middle temporal gyri bilaterally.

**Results:**

The comparison between the two groups revealed several significant differences in the resting-state functional connectivity of the superior and middle temporal gyri in patients with auditory verbal hallucinations as compared to healthy controls. The aberrant connections were focused on the anterior part of the right superior temporal gyrus and the posterior part of the left one, as well as in the posterior division of the right middle temporal gyrus and both anterior and posterior divisions of the left middle temporal gyrus.

**Discussion:**

The observed dysconnectivity between the named subdivisions of the temporal lobe and cortical and subcortical structures suggests that the aberrant connectivity and brain lateralization may be related to the etiopathogenesis of schizophrenia and auditory verbal hallucinations.

## 1 Introduction

Schizophrenia is a disabling mental illness that is estimated to affect approximately 1% of the global population (Velligan and Rao, 2023). This neurodevelopmental psychiatric disorder manifests with a range of symptoms, including positive and negative symptoms, as well as cognitive deficits (Dabiri et al., 2022). Negative symptoms encompass volitional (motivational) impairments, anhedonia, asociality, alogia, as well as emotional disturbances, including affective flattening (Mosolov and Yaltonskaya, 2022). Positive symptoms are defined as conditions that are not present in healthy individuals. These include hallucinations, delusions, disorganized thoughts and speech (Walton et al., 2017). Over 40% of the patients diagnosed with schizophrenia experience auditory verbal hallucinations (AVH), which are one of the most prevalent positive symptoms associated with the condition (Chang et al., 2017; Anhøj et al., 2023). AVH is defined as a phenomenon of perceiving auditory sensations without an external acoustic stimulus (Chyzhyk et al., 2015).

The mechanisms involved in psychotic onset are not entirely elucidated. One widely supported theory suggests that disruptions occurring during brain development, due to either genetic or environmental factors, contribute significantly to an increased vulnerability to psychosis. The neurodevelopmental hypothesis posits that anomalies in the development of the nervous system, especially the establishment of neuronal connections, neuronal migration and transmission of signals, may be an underlying cause of the symptoms associated with schizophrenia (Ricci et al., 2025a). One of the neurotrophic factors that is fundamental to the normal development of the nervous system is glial-derived neurotrophic factor (GDNF). Given its main function in supporting the survival of the motor and dopaminergic neurons, it has been suggested that dysfunctional GDNF may be relevant to the alterations observed in schizophrenia. Additionally, substances of abuse, especially psychostimulants with high dopaminergic activity, prior to the onset of the condition, may act as a triggering factor for the clinical manifestation in individuals with a genetic predisposition to psychosis. This theory represents a pivotal area for investigation concerning the neurotoxic effects of certain substances on the brain and the subsequent restorative actions of GDNF (Ricci et al., 2025a,b).

Functional magnetic resonance imaging (fMRI) is a contemporary, non-invasive technique that is used to examine cognitive processes in both healthy individuals and patients diagnosed with neuropsychiatric disorders. The hemodynamic response reflects the process in which neurons receive additional energy from adjacent capillaries upon activation. This response facilitates an increase in regional cerebral blood flow and oxygen supply and leads to a change in the relative proportions of oxyhemoglobin and deoxyhemoglobin, which can be detected by MR imaging. This imaging technique is referred to as blood oxygen level–dependent (BOLD) contrast imaging. The alteration in the BOLD signal constitutes the fundamental principle of fMRI, which is conventionally employed to generate maps delineating specialized brain regions that are stimulated by specific tasks or external stimuli.

On the other hand, resting-state fMRI (rs-fMRI) is obtained in the absence of external stimulation or a specific task. The underlying principle of rs-fMRI is similarly founded on the BOLD signal fluctuation, which is analogous to that observed in active-task fMRI. The primary focus of rs-fMRI is spontaneous alterations in the BOLD signal, while the subject is at rest. Rs-fMRI data can be analyzed in a variety of ways, with each approach having implications for the information that can be obtained. Functional integration is one of the most common methods. This methodology is employed to ascertain the functional connectivity (FC) between discrete brain regions. FC is defined as the degree of synchrony of the BOLD time-series between distinct cerebral regions. Seed-based FC, also referred to as ROI-based FC, is the process of identifying regions that demonstrate a correlation with the activity observed in a designated seed region. The activation of different brain regions that occurs concurrently suggests that they are engaged in a similar underlying functional process, being interpreted as functionally connected. Conducting this type of analysis necessitates the selection of a seed based on a hypothesis or the findings of previous studies (Lv et al., 2018).

Multiple neuroimaging studies indicate that resting-state functional connectivity (rsFC) differs between individuals diagnosed with schizophrenia and healthy subjects. The alterations include increased rsFC, as well as hypoconnectivity, and have been identified in a range of different brain regions (Weber et al., 2020). Observed disturbances of the FC in patients with schizophrenia and AVH have been most reported in auditory and language related regions (Alderson-Day et al., 2015).

Laterality is a key feature of the human brain. The left hemisphere is widely regarded to be predominant in functions such as handedness and language. Abnormal brain lateralization is proposed to be a risk factor for multiple neuropsychiatric disorders, including schizophrenia (Abuduaini et al., 2023). Reduced brain asymmetry in the auditory cortex has been considered to be linked to schizophrenia. According to the study of Oertel et al. (2010) the decreased lateralization was negatively correlated with the severity of the positive symptoms (as measured by the PANSS), as well as statistically significant negative correlation was established between the reduced laterality and appearance of AVH.

In schizophrenia, abnormal functional lateralization has been observed in the frontal, parietal, temporal and occipital lobes. As noted previously, altered functional lateralization may be linked to impaired inter-hemispheric communication. Studies indicate that the reduced brain lateralization present in individuals diagnosed with schizophrenia may result in a failure of left hemisphere dominance for language (Ribolsi et al., 2009). The reduction in language lateralization may be a contributing factor to the false perception of voices. The decreased functional lateralization of the typical left > right language asymmetry has been found to be significantly correlated with the severity of AVH (Razafimandimby et al., 2007; Chen et al., 2022).

A series of studies examining differences in brain structure and function between AVH and non-AVH (NAVH) patients have identified a number of brain regions implicated in the development of AVH. Among these regions are the inferior frontal gyrus, the superior temporal gyrus (STG), the middle temporal gyrus (MTG), the insula, as well as the basal ganglia (Mo et al., 2024). The STG and the MTG, as components of the language network, have been demonstrated to play a role in auditory and language perception (Friederici and Gierhan, 2013). The majority of studies have reported abnormalities in the rsFC of these regions in patients with schizophrenia experiencing AVH in comparison to healthy individuals (Kubera et al., 2020, Zhang et al., 2017; Chang et al., 2017). These FC alterations encompass enhanced, as well as diminished connectivity between the temporal cortex and different cortical areas, including the frontal, parietal and occipital lobes (Okuneye et al., 2020; Yoon et al., 2015).

Moreover, aberrant FC has also been reported to occur between the temporal lobe and subcortical structures, with the cerebellum, thalamus and basal nuclei being the most frequently mentioned (Damaraju et al., 2014; Hwang et al., 2021; Jia et al., 2023). However, the findings that have been reported were inconsistent, regarding the increased or decreased strength of connectivity within the auditory related areas. Thus, the aim of the present study was to ascertain possible differences in the whole-brain FC and laterality of the STG and the MTG, as seeds in a sample of patients with AVH and a control group of healthy individuals. Based on previous literature, our hypothesis posits that there will be significant lateralized alterations in the rsFC in these regions.

## 2 Materials and methods

### 2.1 Subjects

A total of 110 subjects between the ages of 18 and 61 have been included in the present study and rs-fMRI data was obtained from all of the participants. After a quality assessment of the neuroimaging data, five subjects were excluded. The final sample comprised of 105 participants: 42 patients with AVH and 63 healthy control subjects (HC) who were matched with the schizophrenia patients based on sex and age (±0.7 years).

All participants attended a diagnostic clinical interview with a physician. Psychotic symptoms were assessed using the Positive and Negative Symptom Scale (PANSS). The diagnostic evaluation of the patients revealed that they met the criteria for schizophrenia as defined in the DSMV. The inclusion criterion for the patient group was the presence of severe AVH (P3 > 3). The healthy volunteers have been recruited from the local community. The exclusion criteria for healthy individuals included neurological diseases, psychiatric conditions, and history of brain injury. Intellectual disability was assessed during the initial clinical screening, and individuals presenting with signs of intellectual impairment were excluded from the study. Additional exclusion criteria were established for both patients and healthy controls, including the presence of metal implants or any other contraindication for MRI scans. The participants did not receive any financial compensation. The Ethics Committee of Medical University of Plovdiv approved the study (Protocol No. 1/11.01.2024), and all participants provided written informed consent. The research protocol complied with the 1964 Helsinki Declaration and its subsequent amendments.

### 2.2 MRI scanning procedure

The magnetic resonance scanning procedure was performed on a 3T magnetic resonance imaging system (GE Discovery 750w). The protocol included high resolution structural scan (Sag 3D T1) with the following parameters: slice thickness 1 mm, matrix size 256 × 256, time of relaxation 7.2 ms, time of echo 2.3, angle of flip 12 degrees, voxel size 1 × 1 × 1 mm^3^, and resting-state functional scan – 2D Echo Planar Imaging, slice thickness 3 mms, matrix 64 × 64, repetition time 2 s, echo time 30 ms, 36 slices, angle 90 degrees, voxel size 3.44 × 3.44 × 3.5 mm^3^, a total of 160 slices. All subjects were instructed to maintain composure, to refrain from specific thoughts, and to keep their eyes closed during the scan.

### 2.3 Image processing

The analysis of the functional data was performed using the CONN toolbox (RRID:SCR_009550)^1^ release 21.a (Whitfield-Gabrieli and Nieto-Castanon, 2012), for SPM12 running on MATLAB R2024a for Windows. All images were preprocessed using the default pipeline of the toolbox. The preprocessing included the following steps: functional realignment and unwarp (subject motion estimation and correction); slice-timing correction; outlier identification; direct segmentation and normalization using the Montreal Neurological Institute (MNI) template; and smoothing using spatial convolution with 8-mm full-width-half-maximum 3D Gaussian kernel. Subsequently, the data underwent denoising with the following confound regressors: white matter and cerebrospinal fluid. Then, functional connectivity maps were computed for the superior and middle temporal gyri for each subject. Next, a second-level analysis of Seed-Based Connectivity (SBC) during rest in CONN Toolbox was performed. The significance level was set at *p* < 0.05 (FWE corrected across all comparisons).

### 2.4 Statistical analysis

The socio-demographic data was processed via IBM SPSS Version 28.0 (NY: IBM Corp.). Student’s *t*-test and chi-square test were used for the statistical analysis. The level of statistical significance was set at *p* < 0.05 for all tests.

## 3 Results

### 3.1 Socio-demographic and clinical characteristics

No significant differences were observed in terms of age or sex between patients with schizophrenia and healthy controls. As expected, given the early clinical manifestation of the condition, statistically significant differences were found between the two groups with regard to education. As predicted, the PANSS scores were much higher for the patient group than for the control group (Table 1).

**TABLE 1:** Socio-demographic and clinical characteristics.

Characteristics	AVH (*n* = 42)	HC (*n* = 63)	*P*
Age (mean, SD)	35.3 ± 12.4	36.0 ± 12.5	0.770^a^
Sex (m/f)	26/16	31/32	0.234^b^
^**^Education (primary/secondary/higher)	8/26/6	1/33/29	*0.000^b^
^***^PANSSP3 score (mean, SD)	5.1 ± 0.6	1.0 ± 0.0	*0.000^a^

AVH, auditory verbal hallucinations; HC, healthy controls; *p*, statistical significance; SD, standard deviation.

*^a^*Student’s *t*-test, ^b^χ^2^, test, *p < 0.05, ^**^Two of the patients were excluded from the analysis due to missing data on education, ^***^PANSSP3 score.

### 3.2 Functional connectivity of the superior temporal gyrus

**FIGURE 1:**
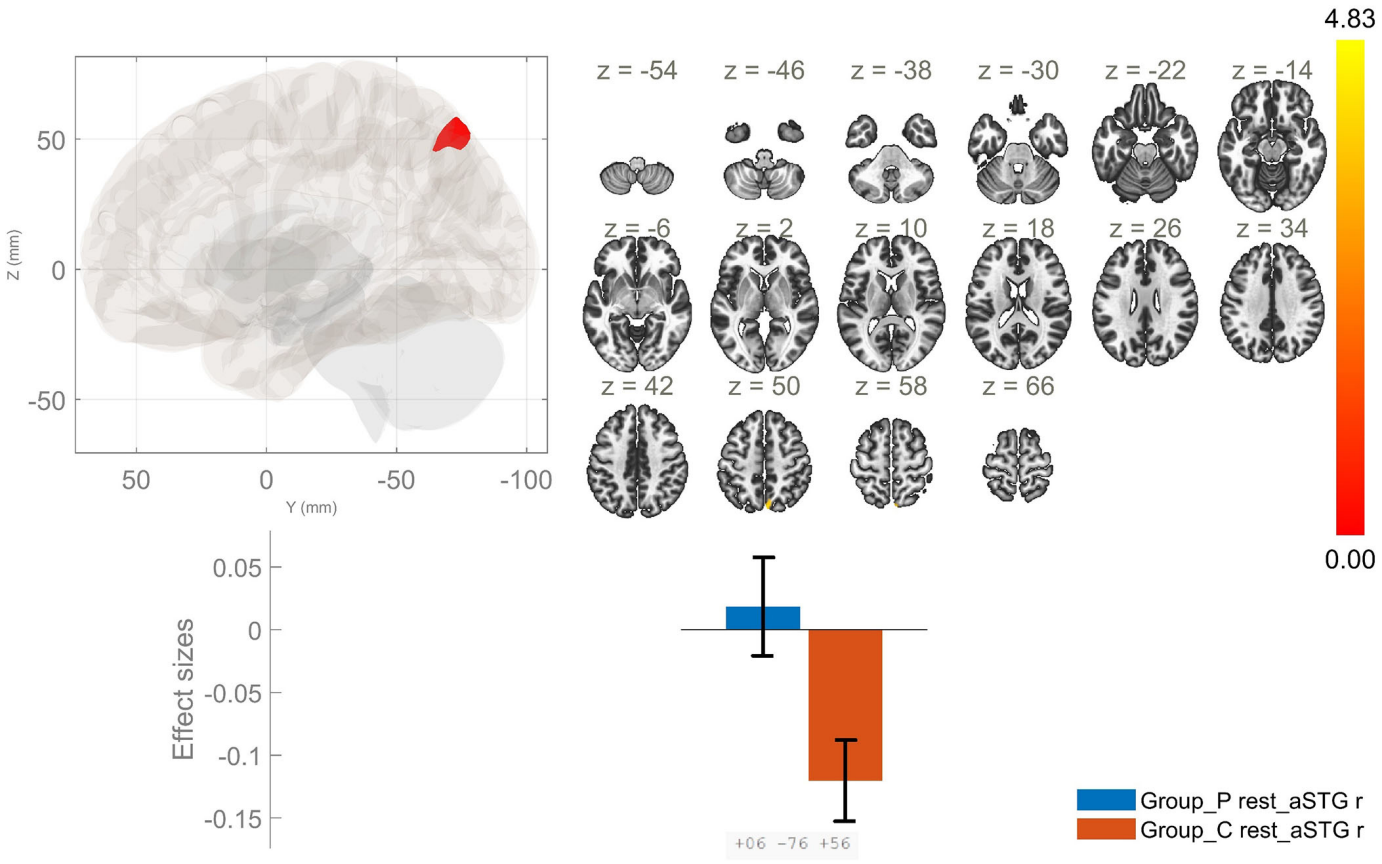
Functional connectivity of the anterior division of the right superior temporal gyrus seed in schizophrenia compared to the HC group and effect sizes for each significant cluster with a cluster threshold *p* < 0.05 cluster-level FWE correction. Increased rsFC between the raSTG and the precuneus (cluster peak coordinates +6, –76, +56) in the patient group in comparison with HC was observed.

**FIGURE 2:**
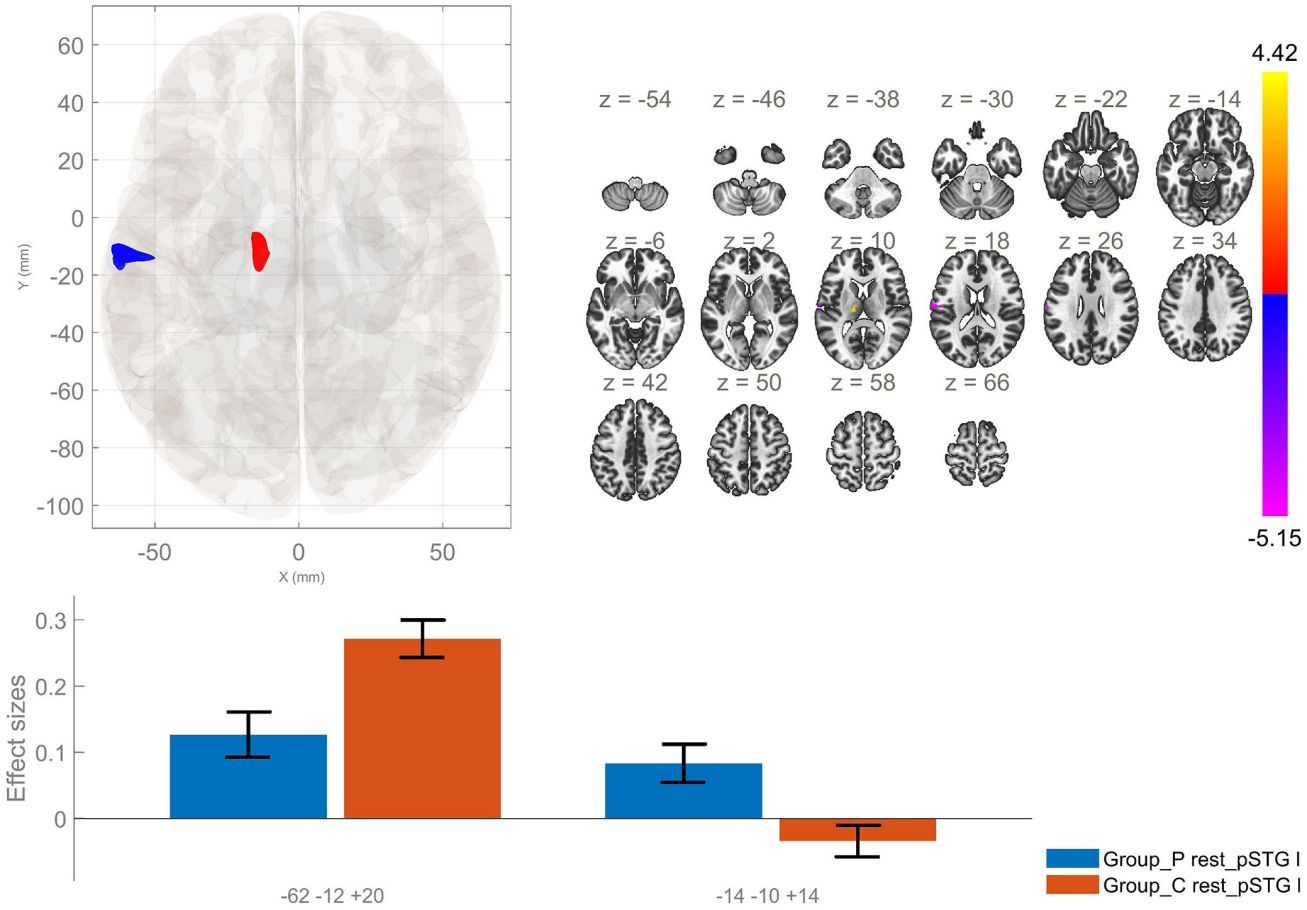
Functional connectivity of the posterior division of the left superior temporal gyrus seed in schizophrenia compared to the HC group and effect sizes for each significant cluster with a cluster threshold *p* < 0.05 cluster-level FWE correction. Reduced rsFC between the left pSTG and the left postcentral gyrus, left central opercular cortex and left planum temporale (cluster peak coordinates –62, –12, +20), as well as increased rsFC between the lpSTG and the left thalamus (–14, –10, +14), in the patient group in comparison with HC, were observed.

The comparison between the patients with schizophrenia and the healthy controls revealed increased FC between the anterior division of the right STG (raSTG) and the precuneus (Figure 1 and Table 2), as well as between the posterior division of the left STG and the left thalamus, in the patient group compared to the healthy controls. Conversely, the patients showed hypoconnectivity between the posterior division of the left STG (lpSTG) and the left postcentral gyrus, left central opercular cortex and left planum temporale (Figure 2 and Table 2). There were no statistically significant rsFC differences for both the anterior division of the left and the posterior division of the right STG.

**TABLE 2:** Between group differences of functional connectivity of the superior temporal gyrus seed.

Seed	Between-group contrast	MNI coordinates x, y, z	Cluster-size	Cluster-threshold (*p* < 0.05, FWE)	Regions within the cluster
Anterior division of right STG	AVH > HC	+6, −76, +56	161	0.004	Precuneus
Posterior division of left STG	AVH > HC	−14, −10, +14	111	0.026	Left thalamus
	HC > AVH	−62, −12, +20	179	0.002	Left postcentral gyrus, left central opercular cortex, left planum temporale

AVH, auditory verbal hallucinations; HC, healthy controls; MNI, Montreal Neurological Institute; FWE, family wise error.

### 3.3 Functional connectivity of the middle temporal gyrus

**FIGURE 3:**
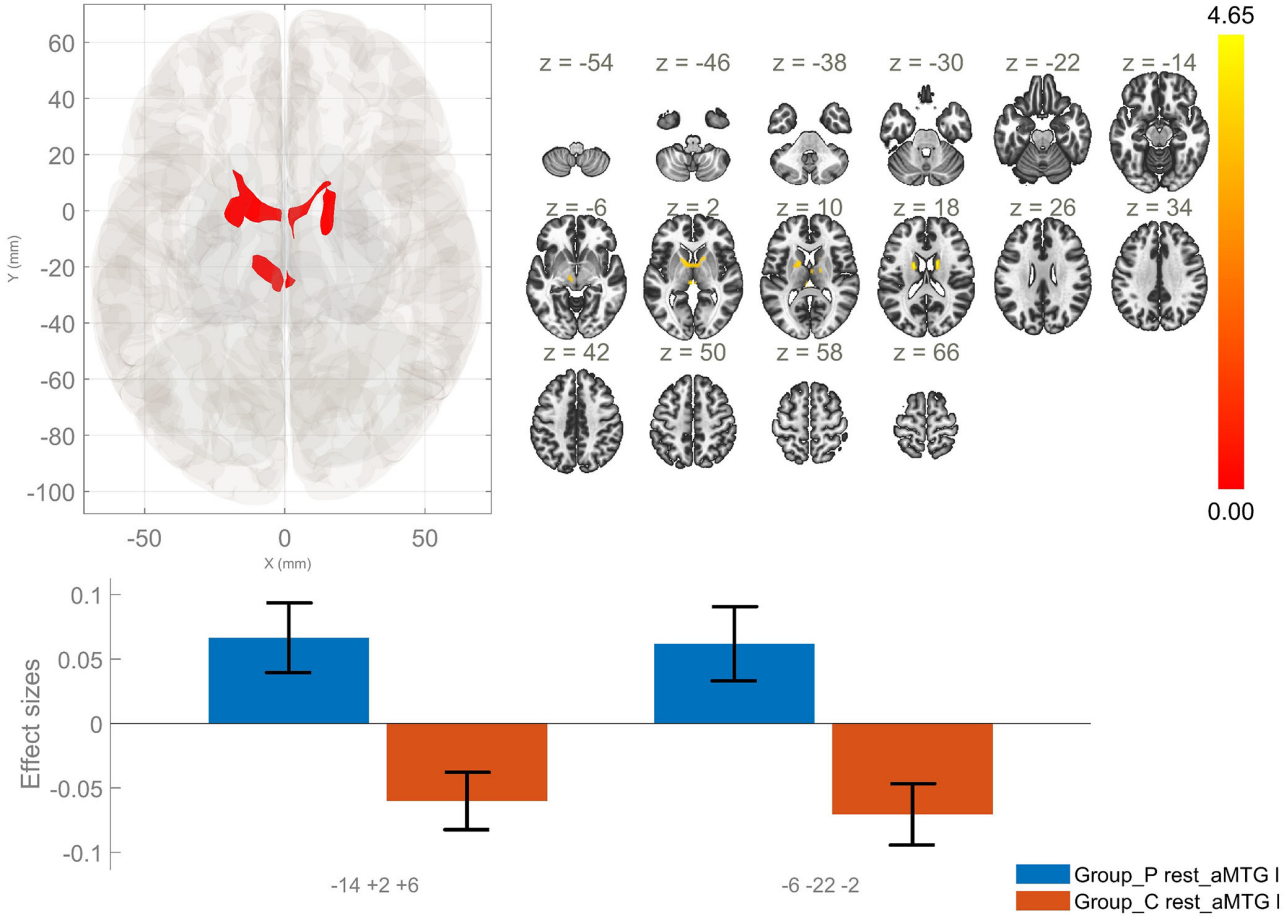
Functional connectivity of the anterior division of the left middle temporal gyrus seed in schizophrenia compared to the HC group and effect sizes for each significant cluster with a cluster threshold *p* < 0.05 cluster-level FWE correction. Increased rsFC between the laMTG and the right thalamus and bilateral caudate nuclei (cluster peak coordinates –14, +2, +6), as well as between laMTG and the left thalamus (–6, –22, –2) in the patient group in comparison with HC were observed.

**FIGURE 4:**
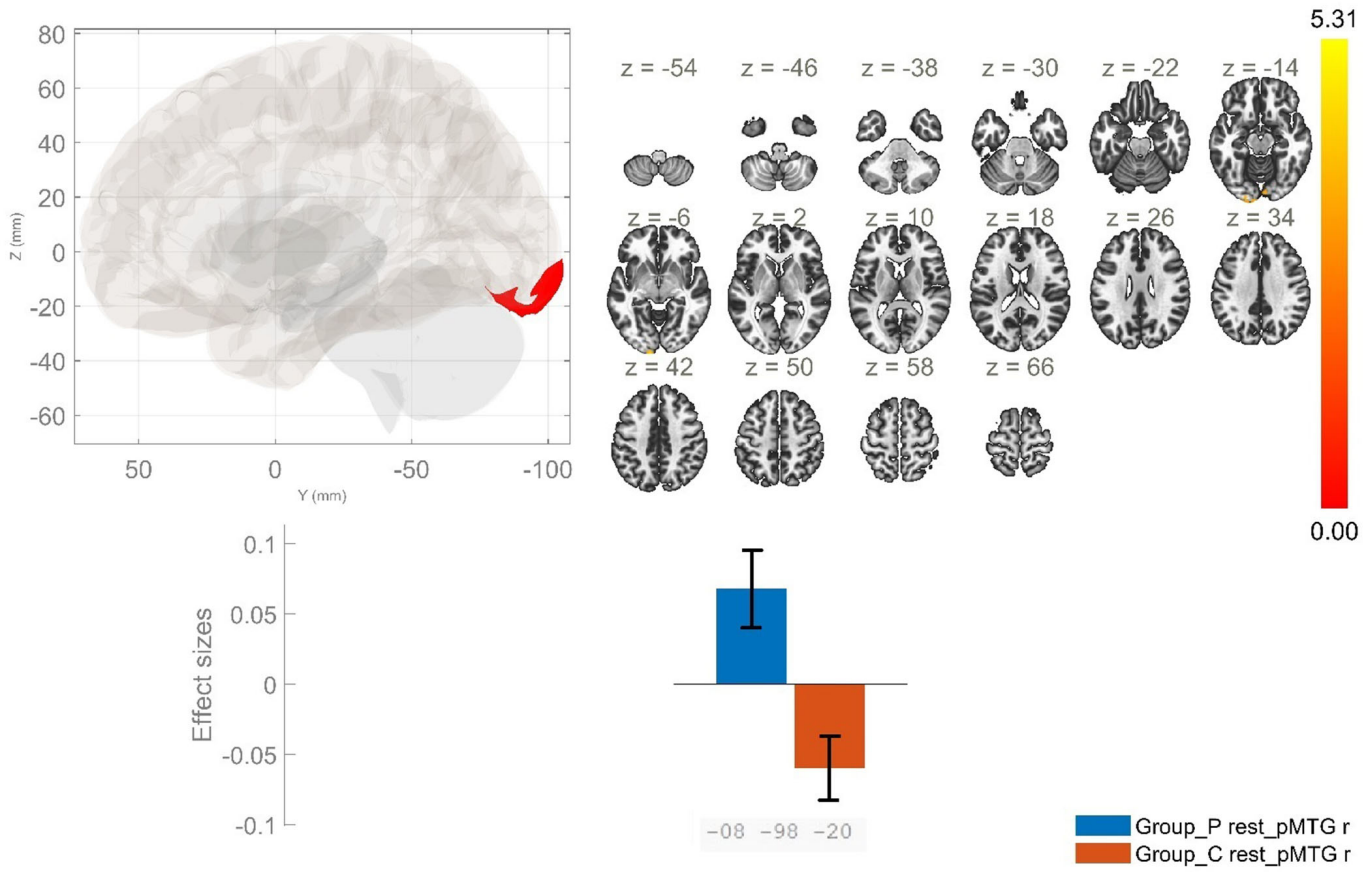
Functional connectivity of the posterior division of the right middle temporal gyrus seed in schizophrenia compared to the HC group and effect sizes for each significant cluster with a cluster threshold *p* < 0.05 cluster-level FWE correction. Increased rsFC between the rpMTG and the occipital pole, as well as the right lingual gyrus (cluster peak coordinates –8, –98, –20) in the patient group in comparison with HC was observed.

The analysis of the rsFC of the anterior division of the right MTG (raMTG) seed demonstrated no statistically significant between-group differences. However, the comparison between the two groups yielded significantly enhanced rsFC between the anterior division of the left MTG (laMTG) and two clusters. The first one involved the right thalamus and bilateral caudate nuclei and the second cluster - comprised of the left thalamus (Figure 3 and Table 3). Furthermore, enhanced rsFC was also observed between the posterior division of the right MTG (rpMTG) and the left occipital pole, as well as in the right lingual gyrus in schizophrenia in contrast to HC (Figure 4 and Table 3).

**TABLE 3:** Between group differences of functional connectivity of the middle temporal gyrus seed.

Seed	Between-group contrast	MNI coordinates x, y, z	Cluster-size	Cluster-threshold (*p* < 0.05, FWE)	Regions within the cluster
Anterior division of left MTG	AVH > HC	−14, +2, +06	510	0.00	Right thalamus, right caudate, left caudate
		−6, −22, −2	192	0.002	Left thalamus
Posterior division of right MTG	AVH > HC	−8, −98, −20	419	0.00	Left occipital pole, right lingual gyrus
Posterior division of left MTG	AVH > HC	−20, +18, −4	118	0.023	Left putamen, left caudate
		+12, −12, +10	106	0.037	Right thalamus
	HC > AVH	−60, −8, +36	99	0.050	Left postcentral gyrus, left precentral gyrus

AVH, auditory verbal hallucinations; HC, healthy controls; MNI, Montreal Neurological Institute; FWE, family wise error.

**FIGURE 5:**
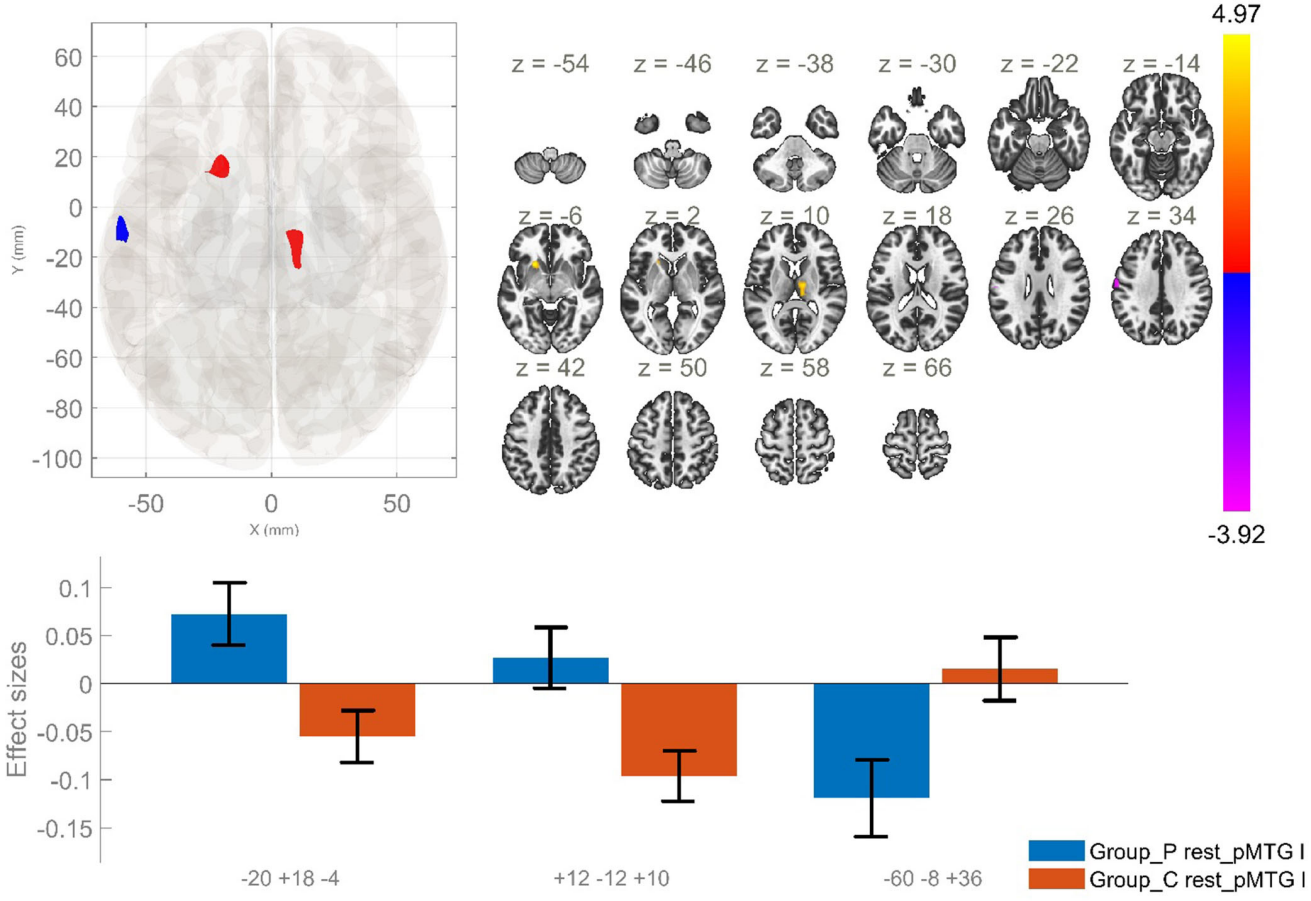
Functional connectivity of the posterior division of the left MTG seed in schizophrenia compared to the HC group and effect sizes for each significant cluster with a cluster threshold *p* < 0.05 cluster-level FWE correction. Increased rsFC between the lpMTG the left putamen and left caudate (cluster peak coordinates –20, +18, –4); increased rsFC between the lpMTG and the right thalamus (+12, –12, +10), as well as reduced rsFC between lpMTG and left precentral and postcentral gyri (–60, –8, +36), in the patient group in comparison with HC, were observed.

The comparison between the patients and healthy individuals showed significantly increased rsFC between the posterior division of the left MTG (lpMTG) and two clusters encompassing the left putamen, left caudate nucleus and the right thalamus. In addition, there was decreased rsFC between the lpMTG and the left postcentral gyrus, as well as between the region of interest and the left precentral gyrus in the patient group as opposed to HC (Figure 5 and Table 3).

## 4 Discussion

The current study identified several significant differences in the rsFC of the STG and MTG in patients with AVH as compared to HC. First, we found an enhanced rsFC between the raSTG and the precuneus in schizophrenia, as opposed to the control group. Next, we identified hyperconnectivity between the lpSTG and the left thalamus, as well as reduced rsFC between this region of interest and the left postcentral gyrus, left central opercular cortex and left planum temporale in the patient group in comparison to HC. Thus, we showed a lateralized impairment in FC of the explored temporal region in AVH with right-sided changes in the anterior division of the STG and left-sided in the posterior one.

Our findings are in accordance with the accumulating evidence from neuroimaging studies suggesting altered FC in the temporal region. In the STG, there are core centers that have been proven to play a regulatory role in both auditory processing, speech generation and perception (Bigler et al., 2007). Abnormal activity in this region has been observed in cases of schizophrenia and AVH (Sommer et al., 2012). In a recent meta-analysis by Ruiz-Torras et al. (2023) hypoconnectivity between the right precentral gyrus and the lSTG was reported. The results of the current study revealed decreased FC between the lpSTG and the central opercular cortex in the AVH group. In previous studies, significant differences in FC between the frontal and temporal cortices have been documented (Yoon et al., 2015; Cui et al., 2017). The research of Hugdahl (2009) has indicated a relationship between the development of AVH and impaired function and integration of bottom-up and top-down brain networks. While AVH are considered as perceptual phenomena (bottom-up), the impact of AVH on inhibitory control, particularly when the individual is overwhelmed by hearing voices, is considered a top-down phenomenon. Hugdahl (2015) proposed that the occurrence of AVH is a result of two distinct processes. Firstly, hyperconnectivity of the temporal cortex leads to the initiation of AVH. Secondly, hypoconnectivity in the frontal cortex is related to an ineffective inhibition of the attentional focus on the voices. Therefore, in order to comprehend the mechanisms of AVH, it is necessary to develop a more profound understanding of the interaction and function of sensory and cognitive brain processes (Kaufmann et al., 2015).

Another study demonstrated that the activation of the STG showed a reduced functional L > R asymmetry in the patient group (Oertel et al., 2010). In the present study, the analysis of the rsFC of the lpSTG exhibited significant between-group differences unlike the rpSTG. These findings may be interpreted as an impairment in the left auditory cortex in schizophrenia and AVH, as compared to healthy individuals.

The planum temporale is a region that is associated with auditory and language processing. This area has been observed to be highly lateralized, with greater volume on the left in right-handed healthy individuals. As already noted, a number of neuroimaging studies have reported a reduction in structural and functional hemispheric asymmetry in individuals diagnosed with schizophrenia. For example, Oertel-Knöchel et al. (2013) reported a reduction in the FC between the planum temporale and cortical (temporal, parietal and limbic) and subcortical areas. These findings suggest the potential involvement of this specific region in the occurrence of AVH (Oertel et al., 2010). Taking into account our findings of hypoconnectivity between the lpSTG and the left planum temporale in the patient group, we suggest that AVH may be related to an impairment of the FC between these two hubs of the auditory network.

The postcentral gyrus is known to contain the primary somatosensory cortex. It has been hypothesized that this region plays a role in integrating information regarding mouth movements into the process of speech perception. A number of studies have documented impairments of the FC between this region and the auditory network. The disruption of integration of sensorimotor processing and the processing of auditory information has been shown to be associated with defective monitoring of inner speech. The disturbance of such language-related abilities in individuals diagnosed with schizophrenia has been demonstrated to result in an elevated risk of experiencing AVH (Chang et al., 2017; Li et al., 2025). In the present study we observed decreased rsFC between the lpSTG and left postcentral gyrus. However, additional research is necessary to explore the implications of the diminished FC between the postcentral gyrus and the auditory network on the clinical symptoms exhibited by individuals diagnosed with schizophrenia.

Our findings also identified increased rsFC between the lpSTG and the left thalamus in the patient group. Similarly, in a study by Cai et al. (2024) hyperconnectivity was observed between the thalamus and the temporal cortex in individuals with schizophrenia compared to HC. Another research by Chen et al. (2020) has also reported enhanced FC between the thalamus and temporal gyri. In contrast, a study by Woodward et al. (2012) reported no group differences in temporal-thalamic connectivity between patients with schizophrenia and HC. Significant alterations were identified between the thalamus and the prefrontal cortex, as well as the motor and somatosensory cortices. One potential explanation for these contradictory results might be the use of large cortical regions as seeds, which may compromise the precision of the analysis at the cortical level (Woodward et al., 2012). Nonetheless, these studies suggest the potential involvement of altered thalamocortical circuits in the etiopathogenesis of schizophrenia. However, further exploration into thalamocortical FC is necessary to validate this hypothesis.

Aberrations in the brain connectivity of the lSTG have been reported in multiple studies. These alterations encompass hyperconnectivity and hypoconnectivity during language task performance, as well as during resting state (Alderson-Day et al., 2016). An interesting finding in the current study was the observed increased rsFC between raSTG and the precuneus. As a key node of the default mode network (DMN), precuneus is implicated in a variety of functions, including episodic memory retrieval, self-processing operations and visuospatial imagery. A substantial disturbance of these functions has been documented in individuals diagnosed with schizophrenia (Kindler et al., 2015).

Multiple studies have reported abnormal rsFC of the DMN in schizophrenia (Alonso-Solís et al., 2015; Hu et al., 2017; Sendi et al., 2021). A study by Forlim et al. (2020) revealed a statistically significant reduction in FC within the DMN in patients diagnosed with schizophrenia compared to healthy individuals. Furthermore, the diminished connectivity in the precuneus exhibited a negative correlation with the negative symptoms. However, most studies have indicated hypoconnectivity within the DMN, increased FC has also been reported. Hyperconnectivity between the temporal cortex (rMTG) and the precuneus was observed in patients with schizophrenia (Li et al., 2025). These findings prompt further inquiry into the functional relationship between the precuneus, a part of the DMN, and the temporal cortex in the pathophysiology of schizophrenia.

The other seed region in our study – MTG showed increased rsFC between the laMTG and two clusters encompassing bilaterally the thalamus and caudate nucleus, in the patient group. In addition, patients demonstrated a statistically significant increase in the rsFC between the lpMTG and subcortical structures, namely putamen – bilaterally, and left caudate nucleus. Conversely, a reduction in the FC between the lpMTG and cortical structures, involving the left postcentral and precentral gyri was characteristic for schizophrenia as opposed to HC. However, a statistically significant enhancement in the rsFC between the rpMTG and a cluster including the left occipital pole and the right lingual gyrus was observed in schizophrenia in comparison to the control group.

Our findings are in accordance with several studies reporting aberrant connectivity between the MTG and the frontal cortex in patients with AVH (Zhang et al., 2017; Stripeikyte et al., 2021). Disturbances in the connectivity strength between the frontal and temporal regions have been linked to the severity of positive symptoms (Ruiz-Torras et al., 2023). Therefore, it is being posited that abnormal frontotemporal connectivity might play a role in the pathophysiology of psychosis. These findings lend support to the hypothesis of functional dysconnectivity as an underlying mechanism in the etiology of schizophrenia.

The current analysis revealed reduced rsFC between the lpMTG and the postcentral gyrus in the AVH group. In a study by Skudlarski et al. (2010) the comparison of rsFC between individuals with schizophrenia and HC yielded hypoconnectivity between lMTG and the postcentral gyrus in the patients group. In addition, the research by Li et al. (2025) indicates a positive correlation between the degree of dysconnectivity between the MTG and the postcentral gyrus and the hallucinations score. Our results are in line with literature and suggest that disrupted connectivity between the temporal and primary somatosensory cortices might contribute to the development of AVH.

Several studies have reported abnormal FC within the sensorimotor network (Berman et al., 2016; Watsky et al., 2018). According to a study by Berman et al. (2016) the presence of positive symptoms is associated with negative interactions between sensorimotor and social-cognitive regions. The results of our study demonstrated disturbed FC between the MTG and key nodes of the sensorimotor network, namely the left precentral and postcentral gyri. Given the fact that the sensorimotor network functions in close conjunction with numerous subnetworks, any irregularities present within the network may also be a contributing factor to impairments of auditory perception.

In a recent report by Wang et al. increased rsFC was observed between the thalamus and bilateral middle temporal lobe in the schizophrenia group as opposed to the control group (Wang et al., 2024). Furthermore, a study by Ferri et al. (2018) posits that the hyperconnectivity between the MTG and the thalamus exhibits a positive correlation with the presence of positive symptoms, specifically hallucinations and delusions. Considering the results of our research of enhanced rsFC between alMTG and the bilateral thalamus, we support the findings of previous studies that the alterations in the thalamo-cortical connectivity may be involved in the development of schizophrenia.

In the current study, enhanced rsFC between the lMTG and caudate was observed. The caudate nucleus is known to be involved in a variety of functions, including linguistic processes and language switching (Mo et al., 2024). Previous studies have reported disturbed connections between the caudate and cortical and subcortical structures, involving the auditory and language network, the sensorimotor network, as well as the basal ganglia and the thalamus in patients with AVH (Mo et al., 2024; Hinkley et al., 2023).

The putamen is a subcortical structure, that has been shown to play a role in speech production (Price, 2010). Research conducted by Hoffman et al. (2011) has demonstrated a correlation between abnormal FC of the putamen in patients with schizophrenia experiencing AVH. In the study of Cui et al. (2016) decreased FC was detected between cortico-striatal-cerebellar networks in individuals with schizophrenia compared to HC. The alterations in question were found to be associated with specific cortical regions, involving the frontal, temporal, and parietal lobes. Furthermore, hypoconnectivity between the left putamen and lMTG was demonstrated in NAVH patients with schizophrenia compared to HC (Cui et al., 2016). Conversely, Zhang et al. (2025) described hyperconnectivity between the rMTG and putamen. Similarly, in our study, the AVH group exhibited hyperconnectivity between the lpMTG and left putamen. In summary, the findings suggest hyperconnectivity between the MTG and the putamen might be involved in the development of AVH. This indicates the necessity of future research focusing on the MTG-putamen connectivity with respect to schizophrenia and AVH.

The results from our study indicate increased rsFC between rpMTG and left occipital pole, as well as the right lingual gyrus. As stated in the systematic review of Tohid et al. (2015) structural and functional abnormalities of the occipital lobe are related to the onset of schizophrenia. The occipital lobe is related to functions such as phonological and semantic modulation. Thus, suggesting potential involvement of aberrant interaction between the occipital lobe and auditory pathways in the pathophysiology of abnormal phonological or semantic processing, which subsequently may result in development of AVH (Tohid et al., 2015). According to a report by Xue et al. (2022) the mechanisms involved in AVH in schizophrenia may be linked to the disrupted dynamic FC between occipital and temporal regions. The results of our study align with those documented in the literature and suggest that aberrant occipito-temporal connectivity may be a contributing factor to the development of AVH.

The findings that have been most consistently reported in the literature are those relating to alterations in connectivity of the left posterior temporal regions, which are involved in speech perception (Alderson-Day et al., 2016). The present study corroborated the findings documented in literature, with the majority of functional connectivity disturbances being detected in the posterior left regions of the STG and MTG.

The present study has some limitations that should be considered. Firstly, the sample size is relatively small. However, the majority of studies have been conducted with a similar number of participants, or fewer. Secondly, the results could be obscured by the impact of antipsychotic drug treatment. Nevertheless, the heterogeneity of medication use (both typical and atypical antipsychotics) reduces the likelihood that a single class of medication drives the observed effects. Another limitation of our study represents the lack of patient subgroup not presenting with AVH. Therefore, the interpretation of the data was executed with utmost caution, in accordance with the findings reported in the extant literature.

### 4.1 Clinical implications

The observed dysconnectivity and altered lateralization in auditory and language-related networks in patients with AVH underscore the relevance of targeting these functional abnormalities in clinical practice. As a translational implication, integrative care approaches combining pharmacological treatment with non-invasive neuromodulation techniques – such as repetitive transcranial magnetic stimulation (rTMS) targeting the left temporoparietal cortex (Yuanjun et al., 2024) – cognitive remediation focused on language and self-monitoring, and sensorimotor integration therapies may offer improved outcomes. Functional connectivity profiles could also aid in individualizing treatment plans and monitoring therapeutic response. Future interdisciplinary models of care integrating neuroimaging biomarkers into early diagnosis and treatment planning may enhance symptom management and recovery in schizophrenia.

## 5 Conclusion

The current study elucidated lateralized differences in the FC of the STG and MTG in AVH. The aberrant connections were focused on the anterior part of the right STG and the posterior part of the left STG, as well as in the posterior division of the right MTG and both anterior and posterior division of the left MTG. The observed dysconnectivity between the named subdivisions of the temporal lobe and the noted cortical and subcortical structures suggests that the aberrant connectivity and brain lateralization may be related to the etiopathogenesis of schizophrenia and AVH. These findings support the dysconnectivity hypothesis as a predominant pathophysiological mechanism in schizophrenia and suggest specific brain regions for targeted individualized treatment.

## Data Availability

The raw data supporting the conclusions of this article will be made available by the authors, without undue reservation.

## Footnotes

1
www.nitrc.org/projects/conn

## References

[B1] Abuduaini Y. Pu Y. Thompson P. M. Kong X. (2023). Significant heterogeneity in structural asymmetry of the habenula in the human brain: A systematic review and meta-analysis. *Hum. Brain Mapp.* 44 4165–4182. 10.1002/hbm.26337 37195040 PMC10258539

[B2] Alderson-Day B. Diederen K. Fernyhough C. Ford J. M. Horga G. Margulies D. S. (2016). Auditory hallucinations and the brain’s resting-state networks: Findings and methodological observations. *Schizophrenia Bull.* 42 1110–1123. 10.1093/schbul/sbw078 27280452 PMC4988751

[B3] Alderson-Day B. McCarthy-Jones S. Fernyhough C. (2015). Hearing voices in the resting brain: A review of intrinsic functional connectivity research on auditory verbal hallucinations. *Neurosci. Biobehav. Rev.* 55 78–87. 10.1016/j.neubiorev.2015.04.016 25956256 PMC5901708

[B4] Alonso-Solís A. Vives-Gilabert Y. Grasa E. Portella M. J. Rabella M. Sauras R. B. (2015). Resting-state functional connectivity alterations in the default network of schizophrenia patients with persistent auditory verbal hallucinations. *Schizophrenia Res.* 161 261–268. 10.1016/j.schres.2014.10.047 25468173

[B5] Anhøj S. Ebdrup B. Nielsen M. Ø Antonsen P. Glenthøj B. Rostrup E. (2023). Functional connectivity between auditory and medial temporal lobe networks in antipsychotic-naïve patients with first-episode schizophrenia predicts the effects of dopamine antagonism on auditory verbal hallucinations. *Biol. Psychiatry Global Open Sci.* 4 308–316. 10.1016/j.bpsgos.2023.06.003 38298804 PMC10829637

[B6] Berman R. A. Gotts S. J. Mcadams H. M. Greenstein D. Lalonde F. Clasen L. (2016). Disrupted sensorimotor and social–cognitive networks underlie symptoms in childhood-onset schizophrenia. *Brain* 139 276–291. 10.1093/brain/awv306 26493637 PMC4719706

[B7] Bigler E. D. Mortensen S. Neeley E. S. Ozonoff S. Krasny L. Johnson M. (2007). Superior temporal gyrus, language function, and autism. *Dev. Neuropsychol.* 31 217–238. 10.1080/87565640701190841 17488217

[B8] Cai J. Xie M. Liang S. Gong J. Deng W. Guo W. (2024). Dysfunction of thalamocortical circuits in early-onset schizophrenia. *Cereb. Cortex* 34:bhae313. 10.1093/cercor/bhae313 39106176

[B9] Chang X. Collin G. Xi Y. Cui L. Scholtens L. H. Sommer I. E. (2017). Resting-state functional connectivity in medication-naïve schizophrenia patients with and without auditory verbal hallucinations: A preliminary report. *Schizophrenia Res.* 188 75–81. 10.1016/j.schres.2017.01.024 28130005

[B10] Chen C. Huang H. Qin X. Zhang L. Rong B. Wang G. (2022). Reduced inter-hemispheric auditory and memory-related network interactions in patients with schizophrenia experiencing auditory verbal hallucinations. *Front. Psychiatry* 13:956895. 10.3389/fpsyt.2022.956895 35990049 PMC9381966

[B11] Chen M. H. Chang W. C. Bai Y. M. Huang K. L. Tu P. C. Su T. P. (2020). Cortico-thalamic dysconnection in early-stage schizophrenia: A functional connectivity magnetic resonance imaging study. *Eur. Arch. Psychiatry Clin. Neurosci.* 270 351–358. 10.1007/s00406-019-01003-2 30953128

[B12] Chyzhyk D. Graña M. Öngür D. Shinn A. K. (2015). Discrimination of schizophrenia auditory hallucinators by machine learning of resting-state functional MRI. *Int. J. Neural Syst.* 25:1550007. 10.1142/s0129065715500070 25753600 PMC4787625

[B13] Cui L. B. Liu K. Li C. Wang L. X. Guo F. Tian P. (2016). Putamen-related regional and network functional deficits in first-episode schizophrenia with auditory verbal hallucinations. *Schizophrenia Res.* 173 13–22. 10.1016/j.schres.2016.02.039 26995674

[B14] Cui L.-B. Liu L. Guo F. Chen Y.-C. Chen G. Xi M. (2017). Disturbed brain activity in resting-state networks of patients with first-episode schizophrenia with auditory verbal hallucinations: A cross-sectional functional MR imaging study. *Radiology* 283 810–819. 10.1148/radiol.2016160938 28045645

[B15] Dabiri M. Dehghani Firouzabadi F. Yang K. Barker P. B. Lee R. R. Yousem D. M. (2022). Neuroimaging in schizophrenia: A review article. *Front. Neurosci.* 16:1042814. 10.3389/fnins.2022.1042814 36458043 PMC9706110

[B16] Damaraju E. Allen E. A. Belger A. Ford J. M. McEwen S. Mathalon D. H. (2014). Dynamic functional connectivity analysis reveals transient states of dysconnectivity in schizophrenia. *NeuroImage Clin.* 5 298–308. 10.1016/j.nicl.2014.07.003 25161896 PMC4141977

[B17] Ferri J. Ford J. M. Roach B. J. Turner J. A. (2018). Resting-state thalamic dysconnectivity in schizophrenia and relationships with symptoms. *Psychol. Med.* 48 2492–2499. 10.1017/S003329171800003X 29444726 PMC12094034

[B18] Forlim C. G. Klock L. Bächle J. Stoll L. Giemsa P. Fuchs M. (2020). Reduced resting-state connectivity in the precuneus is correlated with apathy in patients with schizophrenia. *Sci. Rep.* 10:2616. 10.1038/s41598-020-59393-6 32054907 PMC7018974

[B19] Friederici A. D. Gierhan S. M. (2013). The language network. *Curr. Opin. Neurobiol.* 23 250–254. 10.1016/j.conb.2012.10.002 23146876

[B20] Hinkley L. B. N. Haas S. S. Cheung S. W. Nagarajan S. S. Subramaniam K. (2023). Reduced neural connectivity in the caudate anterior head predicts hallucination severity in schizophrenia. *Schizophrenia Res.* 261 1–5. 10.1016/j.schres.2023.08.030 37678144 PMC10878029

[B21] Hoffman R. E. Fernandez T. Pittman B. Hampson M. (2011). Elevated functional connectivity along a corticostriatal loop and the mechanism of auditory/verbal hallucinations in patients with schizophrenia. *Biol. Psychiatry* 69 407–414. 10.1016/j.biopsych.2010.09.050 21145042 PMC3039042

[B22] Hu M. L. Zong X. F. Mann J. J. Zheng J. J. Liao Y. H. Li Z. C. (2017). A review of the functional and anatomical default mode network in schizophrenia. *Neurosci. Bull.* 33 73–84. 10.1007/s12264-016-0090-1 27995564 PMC5567552

[B23] Hugdahl K. (2009). “Hearing voices”: Auditory hallucinations as failure of top-down control of bottom-up perceptual processes. *Scand. J. Psychol.* 50 553–560. 10.1111/j.1467-9450.2009.00775.x 19930254

[B24] Hugdahl K. (2015). Auditory hallucinations: A review of the ERC “VOICE” project. *World J. Psychiatry* 5 193–209. 10.5498/wjp.v5.i2.193 26110121 PMC4473491

[B25] Hwang M. Roh Y. S. Talero J. Cohen B. M. Baker J. T. Brady R. O. (2021). Auditory hallucinations across the psychosis spectrum: Evidence of dysconnectivity involving cerebellar and temporal lobe regions. *NeuroImage Clin.* 32:102893. 10.1016/j.nicl.2021.102893 34911197 PMC8636859

[B26] Jia Y. Jariwala N. Hinkley L. B. N. Nagarajan S. Subramaniam K. (2023). Abnormal resting-state functional connectivity underlies cognitive and clinical symptoms in patients with schizophrenia. *Front. Hum. Neurosci.* 17:1077923. 10.3389/fnhum.2023.1077923 36875232 PMC9976937

[B27] Kaufmann T. Skåtun K. C. Alnæs D. Doan N. T. Duff E. P. Tønnesen S. (2015). Disintegration of sensorimotor brain networks in schizophrenia. *Schizophrenia Bull.* 41 1326–1335. 10.1093/schbul/sbv060 25943122 PMC4601711

[B28] Kindler J. Jann K. Homan P. Hauf M. Walther S. Strik W. (2015). Static and dynamic characteristics of cerebral blood flow during the resting state in schizophrenia. *Schizophrenia Bull.* 41 163–170. 10.1093/schbul/sbt180 24327756 PMC4266282

[B29] Kubera K. M. Wolf N. D. Rashidi M. Hirjak D. Northoff G. Schmitgen M. M. (2020). Functional decoupling of language and self-reference networks in patients with persistent auditory verbal hallucinations. *Neuropsychobiology* 79 345–351. 10.1159/000507630 32485705

[B30] Li H. Zhang W. Song H. Zhuo L. Yao H. Sun H. (2025). Altered temporal lobe connectivity is associated with psychotic symptoms in drug-naïve adolescent patients with first-episode schizophrenia. *Eur. Child Adolesc. Psychiatry* 34 237–247. 10.1007/s00787-024-02485-9 38832962

[B31] Lv H. Wang Z. Tong E. Williams L. M. Zaharchuk G. Zeineh M. (2018). resting-state functional MRI: Everything that nonexperts have always wanted to know. *Am. J. Neuroradiol.* 39 1390–1399. 10.3174/ajnr.a5527 29348136 PMC6051935

[B32] Mo F. Zhao H. Li Y. Cai H. Song Y. Wang R. (2024). Network localization of state and trait of auditory verbal hallucinations in schizophrenia. *Schizophrenia Bull.* 50 1326–1336. 10.1093/schbul/sbae020 38401526 PMC11548935

[B33] Mosolov S. N. Yaltonskaya P. A. (2022). Primary and secondary negative symptoms in schizophrenia. *Front. Psychiatry* 12:766692. 10.3389/fpsyt.2021.766692 35046851 PMC8761803

[B34] Oertel V. Knöchel C. Rotarska-Jagiela A. Schönmeyer R. Lindner M. Van De Ven V. (2010). Reduced laterality as a trait marker of schizophrenia—Evidence from structural and functional neuroimaging. *J. Neurosci.* 30 2289–2299. 10.1523/jneurosci.4575-09.2010 20147555 PMC6634045

[B35] Oertel-Knöchel V. Knöchel C. Matura S. Prvulovic D. Linden D. E. van de Ven V. (2013). Reduced functional connectivity and asymmetry of the planum temporale in patients with schizophrenia and first-degree relatives. *Schizophrenia Res.* 147 331–338. 10.1016/j.schres.2013.04.024 23672819

[B36] Okuneye V. T. Meda S. Pearlson G. D. Clementz B. A. Keshavan M. S. Tamminga C. A. (2020). Resting state auditory-language cortex connectivity is associated with hallucinations in clinical and biological subtypes of psychotic disorders. *NeuroImage Clin.* 27:102358. 10.1016/j.nicl.2020.102358 32745995 PMC7398970

[B37] Price C. J. (2010). The anatomy of language: A review of 100 fMRI studies published in 2009. *Ann. N. Y. Acad. Sci.* 1191 62–88. 10.1111/j.1749-6632.2010.05444.x 20392276

[B38] Razafimandimby A. Maïza O. Hervé P. Y. Lecardeur L. Delamillieure P. Brazo P. (2007). Stability of functional language lateralization over time in schizophrenia patients. *Schizophrenia Res.* 94 197–206. 10.1016/j.schres.2007.04.011 17570644

[B39] Ribolsi M. Koch G. Magni V. Di Lorenzo G. Rubino I. A. Siracusano A. (2009). Abnormal brain lateralization and connectivity in schizophrenia. *Rev. Neurosci.* 20 61–70. 10.1515/revneuro.2009.20.1.61 19526734

[B40] Ricci V. De Berardis D. Martinotti G. Maina G. (2025a). Glial derived neurotrophic factor and schizophrenia spectrum disorders: A scoping review. *Curr. Neuropharmacol.* 23 564–578. 10.2174/011570159X340124241205095729 39679463 PMC12163499

[B41] Ricci V. Sarni A. De Berardis D. Fraccalini T. Martinotti G. Maina G. (2025b). Symptomatic predictors of suicidal behavior in early psychosis: Systematic review. *J. Psychiatric Pract.* 31 125–138. 10.1097/PRA.0000000000000860 40440668

[B42] Ruiz-Torras S. Gudayol-Ferré E. Fernández-Vazquez O. Cañete-Massé C. Peró-Cebollero M. Guàrdia-Olmos J. (2023). Hypoconnectivity networks in schizophrenia patients: A voxel-wise meta-analysis of Rs-fMRI. *Int. J. Clin. Health Psychol.* 23:100395. 10.1016/j.ijchp.2023.100395 37533450 PMC10392089

[B43] Sendi M. S. E. Zendehrouh E. Ellis C. A. Liang Z. Fu Z. Mathalon D. H. (2021). Aberrant dynamic functional connectivity of default mode network in schizophrenia and links to symptom severity. *Front. Neural Circuits* 15:649417. 10.3389/fncir.2021.649417 33815070 PMC8013735

[B44] Skudlarski P. Jagannathan K. Anderson K. Stevens M. C. Calhoun V. D. Skudlarska B. A. (2010). Brain connectivity is not only lower but different in schizophrenia: A combined anatomical and functional approach. *Biol. Psychiatry* 68 61–69. 10.1016/j.biopsych.2010.03.035 20497901 PMC2900394

[B45] Sommer I. E. Clos M. Meijering A. L. Diederen K. M. J. Eickhoff S. B. (2012). Resting state functional connectivity in patients with chronic hallucinations. *PLoS One* 7:e43516. 10.1371/journal.pone.0043516 22970130 PMC3435327

[B46] Stripeikyte G. Potheegadoo J. Progin P. Rognini G. Blondiaux E. Salomon R. (2021). Fronto-temporal disconnection within the presence hallucination network in psychotic patients with passivity experiences. *Schizophrenia Bull.* 47 1718–1728. 10.1093/schbul/sbab031 33823042 PMC8530400

[B47] Tohid H. Faizan M. Faizan U. (2015). Alterations of the occipital lobe in schizophrenia. *Neurosciences* 20 213–224. 10.17712/nsj.2015.3.20140757 26166588 PMC4710336

[B48] Velligan D. I. Rao S. (2023). The epidemiology and global burden of schizophrenia. *J. Clin. Psychiatry* 84:MS21078COM5. 10.4088/jcp.ms21078com5 36652681

[B49] Walton E. Hibar D. P. Van Erp T. G. M. Potkin S. G. Roiz-Santiañez R. Crespo-Facorro B. (2017). Positive symptoms associate with cortical thinning in the superior temporal gyrus via the ENIGMA Schizophrenia consortium. *Acta Psychiatr. Scand.* 135 439–447. 10.1111/acps.12718 28369804 PMC5399182

[B50] Wang L. N. Lin S. Tian L. Wu H. Jin W. Q. Wang W. (2024). Subregional thalamic functional connectivity abnormalities and cognitive impairments in first-episode schizophrenia. *Asian J. Psychiatry* 96:104042. 10.1016/j.ajp.2024.104042 38615577

[B51] Watsky R. E. Gotts S. J. Berman R. A. McAdams H. M. Zhou X. Greenstein D. (2018). Attenuated resting-state functional connectivity in patients with childhood- and adult-onset schizophrenia. *Schizophrenia Res.* 197 219–225. 10.1016/j.schres.2018.01.003 29310911 PMC6035109

[B52] Weber S. Johnsen E. Kroken R. A. Løberg E.-M. Kandilarova S. Stoyanov D. (2020). Dynamic functional connectivity patterns in schizophrenia and the relationship with hallucinations. *Front. Psychiatry* 11:227. 10.3389/fpsyt.2020.00227 32300313 PMC7145395

[B53] Whitfield-Gabrieli S. Nieto-Castanon A. (2012). CONN: A functional connectivity toolbox for correlated and anticorrelated brain networks. *Brain Connect.* 2, 125–141. 10.1089/brain.2012.0073 22642651

[B54] Woodward N. D. Karbasforoushan H. Heckers S. (2012). Thalamocortical dysconnectivity in Schizophrenia. *Am. J. Psychiatry* 169 1092–1099. 10.1176/appi.ajp.2012.12010056 23032387 PMC3810300

[B55] Xue K. Chen J. Wei Y. Chen Y. Han S. Wang C. (2022). Altered dynamic functional connectivity of auditory cortex and medial geniculate nucleus in first-episode, drug-naïve schizophrenia patients with and without auditory verbal hallucinations. *Front. Psychiatry* 13:963634. 10.3389/fpsyt.2022.963634 36159925 PMC9489854

[B56] Yoon Y. B. Yun J.-Y. Jung W. H. Cho K. I. K. Kim S. N. Lee T. Y. (2015). Altered fronto-temporal functional connectivity in individuals at ultra-high-risk of developing psychosis. *PLoS One* 10:e0135347. 10.1371/journal.pone.0135347 26267069 PMC4534425

[B57] Yuanjun X. Guan M. Zhang T. Ma C. Wang L. Lin X. (2024). Targeting auditory verbal hallucinations in schizophrenia: Effective connectivity changes induced by low-frequency rTMS. *Transl. Psychiatry* 14:393. 10.1038/s41398-024-03106-4 39341819 PMC11438995

[B58] Zhang L. Guo L. Liu X. Han J. Zhu Y. Ma C. (2025). Low-frequency rTMS modulates small-world network properties in an AVH-related brain network in schizophrenia. *Front. Psychiatry* 16:1578072. 10.3389/fpsyt.2025.1578072 40303447 PMC12037504

[B59] Zhang L. Li B. Wang H. Li L. Liao Q. Liu Y. (2017). Decreased middle temporal gyrus connectivity in the language network in schizophrenia patients with auditory verbal hallucinations. *Neurosci. Lett.* 653 177–182. 10.1016/j.neulet.2017.05.042 28572034

